# Protective Effects and Mechanism of *Heracleum moellendorffii Hance* on Alcohol-Induced Cognitive Decline in Mice

**DOI:** 10.3390/ijms25158526

**Published:** 2024-08-05

**Authors:** Woohee Park, Yunna Kim, Seung-Hun Cho

**Affiliations:** 1 Department of Clinical Korean Medicine, Graduate School, Kyung Hee University, Seoul 02447, Republic of Korea; parkwoohee31@naver.com; 2 Department of Neuropsychiatry, College of Korean Medicine, Kyung Hee University Medical Center, Kyung Hee University, Seoul 02447, Republic of Korea; yunna.anna.kim@khu.ac.kr; 3 Research Group of Neuroscience, East-West Medical Research Institute, WHO Collaborating Center, Kyung Hee University, Seoul 02447, Republic of Korea

**Keywords:** *Heracleum moellendorffii Hance*, alcohol-related dementia, cognitive impairment, memory, BDNF, CREB

## Abstract

Chronic and continuous alcohol consumption increases the risk of cognitive decline and may lead to alcohol-related dementia. We investigated the potential of *Heracleum moellendorffii Hance* root extract (HME) for treating alcohol-related cognitive impairment. Behavioral tests evaluated the effects of HME on cognitive function and depression. Changes in hippocampus and liver tissues were evaluated by Western blotting and H&E staining. The group treated with HME 200 mg/kg showed a significant increase in spontaneous alternation in Y-maze and a decrease in immobility in a forced swimming test (FST) compared to the vehicle-treated group. These results suggest that HME can restore memory deficits and reverse depressive symptoms caused by chronic alcohol consumption. The HME-treated group also upregulated brain-derived neurotrophic factor (BDNF), phosphorylated extracellular signal-regulated kinase 1/2 (ERK1/2), and phosphorylated cAMP response element-binding protein (CREB) in the hippocampus. Additionally, it reduced lipid vacuolation in the liver and increased the expression of aldehyde dehydrogenase 1 (ADH1). The administration of HME improves cognitive impairment and reverses depressive symptoms due to alcohol consumption, restoring neural plasticity in the hippocampus and alcohol metabolism in the liver. These findings suggest that HME is a promising treatment for alcohol-related brain disorders. Molecular mechanisms underlying the therapeutic effects of HME and its active ingredients should be investigated further.

## 1. Introduction

Dementia is a disease that affects memory, cognitive function, emotional and behavioral responses, and other symptoms and is becoming increasingly prevalent in our community [1]. Acute alcohol ingestion can cause blackouts, while prolonged exposure can lead to alcohol-related dementia (ARD), which can impact cognitive functions [2]. The incidence per 100,000 person-years of ARD was estimated to be 8.2 in men and 2.1 in women [3]. It has been reported that an increase of 7 units per week in alcohol consumption leads to a 17% increase in the risk of dementia [4].

In alcohol-induced neurodegeneration and cognitive decline, the reduction in extracellular signal regulatory kinase (ERK1/2) and c-AMP-response element-binding protein (CREB) largely inhibits brain-derived neurotrophic factor (BDNF) [5]. This pathway has been suggested to mitigate cognitive decline associated with alcohol consumption [6]. The factors BDNF, ERK1/2, and CREB promote adult hippocampal neurogenesis, which contributes to learning and memory. BDNF increases cell birth, survival, and maturation, and this effect is regulated by the ERK pathway, while CREB regulates the expression of genes involved in neurogenesis [7]. Alcohol exposure can affect neurogenesis and memory by modulating BDNF, ERK1/2, and CREB signaling in the brain [8]. Excessive drinking, a prevalent cause of neurological problems, is associated with direct toxicity and nutritional deficiencies resulting from ingested alcohol and its metabolite, acetaldehyde [9]. Alcohol dehydrogenase is the primary enzyme responsible for the liver’s metabolism of ethanol, resulting in the production of acetaldehyde [10]. Acetaldehyde can cause DNA damage in various parts of the body, including the brain, by reducing CREB levels [11]. Although moderate alcohol concentration has only a slight influence on speed and accuracy, exposure to acetaldehyde after acute intravenous alcohol appears to have a negative impact on sustained attention and working memory in human subjects [12].

The main difference between ARD and Alzheimer’s disease, which is the most common form of dementia, is that in ARD, the cognitive decline does not progress further, and may even improve if alcohol consumption is ceased [13]. Therefore, early intervention is crucial in preventing the deterioration of ARD. One study found that memantine, a treatment for severe Alzheimer’s disease, reduced the desire for alcohol consumption and improved cognitive function in patients with ARD [14].

However, for individuals at high risk or in the early stages, safer and more effective natural products may be a suitable alternative. Considering the caution surrounding the use of memantine in patients with liver disorders and renal dysfunction, it may be worthwhile to explore other options for prevention and early treatment, including natural products that have been used for a long time and have been shown to be safe [15]. Previously, a study showed that red ginseng extract was effective against alcohol-induced neurotoxicity by inhibiting cell self-destruction, increasing neuronal cell survival, and promoting nerve regeneration [16].

*Heracleum moellendorffii* extract *(HME)* is an edible wild herb of the Umbelliferae family. It is mainly distributed in Korea, China and Japan. Traditional Korean medicine has utilized HME due to its exceptional effects on neuralgia and anti-inflammatory properties [17,18]. HME contains several flavonoids, including skimmin, monoterpenoids, sesquiterpenoids, isopimpinellin, and polyacetylenes [17]. It has been noted that HME is effective for skin diseases, diabetes, fever, pain, and arthritis. HME has been reported to have immunomodulatory, anti-inflammatory, antiviral, and anti-obesity effects [17,19,20,21]. HME may potentially improve memory and cognitive impairment and reverse depression, as the neuroprotective and lipid metabolism effects are relevant to alcohol-related cognitive problems. In addition, HME contains furocoumarin, which has been reported to inhibit acetylcholine esterase [22].

In this study, the effect of HME was examined in an animal model of ARD, which showed cognitive decline induced by continuous alcohol intake and binge drinking, with reference to previous studies [23]. We hypothesize that HME may mitigate cognitive and mood changes induced by chronic alcohol intake through its regulatory effects on the hippocampus and liver. The effect of HME on cognition and mood changes after chronic alcohol intake and its regulatory effects on the hippocampus and liver were investigated.

## 2. Results

### 2.1. Effect of Long-Term Alcohol Exposure and HME Administration on Baseline Profiles of Mice

**Figure 1:**
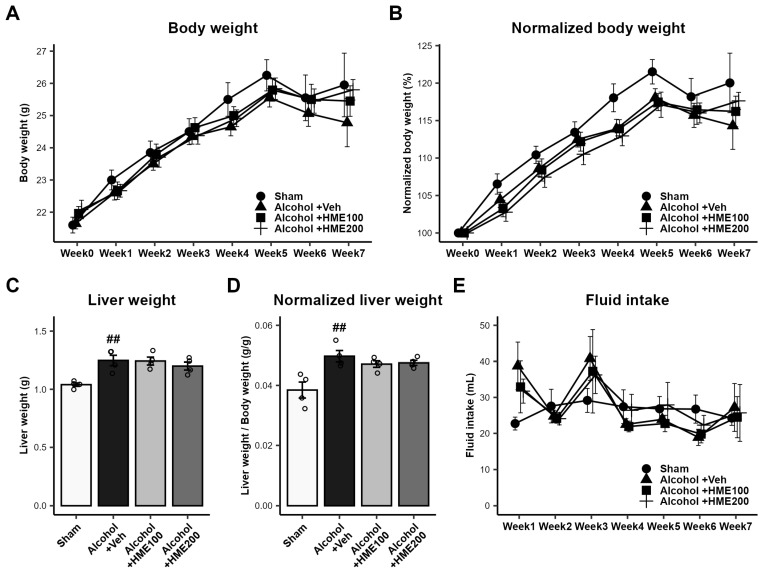
The effect of long-term alcohol intake and HME treatment on body weight and liver weight in the four groups of mice. (**A**) Body weight and (**B**) body weight normalized to the baseline weight in Week 0. HME did not affect the body weight of mice. n = 10/group. (**C**) Liver weight of mice and (**D**) liver weight normalized to the body weight. The values are represented as mean ± SEM. n = 4/group. (**E**) The weekly fluid intake per mouse. n = 10/group. The values are represented as mean ± SEM. ^##^ *p* < 0.001, compared with the sham group. Sham, sham control; Alcohol + Veh, EtOH administration and vehicle treatment; Alcohol + HME100, EtOH administration and HME 100 mg/kg treatment; Alcohol + HME200, EtOH administration and HME 200 mg/kg treatment; EtOH, ethanol; HME, *Heracleum moellendorfii* Hance extract; Veh, vehicle.

There was no difference in the mean body weight and the body weight normalized to baseline weight in Week 0 among the four groups of mice until the third week of the experiment (sham group vs. 10% EtOH intake in the other three groups). However, after the consumption of 20% EtOH from the fourth week of the experiment, the mean weight of the sham group of mice differed from the mean weight of the three alcohol-consuming groups. However, after the administration of HME from the fifth week, the difference between the sham group and the two HME-treated groups decreased. In contrast, the control group that consumed alcohol (Alcohol + Veh group) showed little weight gain. At week 7, the weight of the two HME groups increased slowly compared to the sham group (Figure 1A,B).

Continuous alcohol consumption significantly increased liver weight (*p* < 0.01). Notably, HME treatment at doses of 100 mg/kg and 200 mg/kg did not affect the change in liver weight (*p* > 0.05, versus Alcohol + Veh; Figure 1C,D).

The sham group, which consumed water, showed a constant volume of water consumption throughout the study. The groups with EtOH intake exhibited slightly higher consumption in the first week and the third week, but there was no significant difference in the sham group in the other weeks (Figure 1E).

### 2.2. Effect of HME Treatment on Locomotor Activity in Alcohol-Ingested Mice in the Open Field Test (OFT)

**Figure 2:**
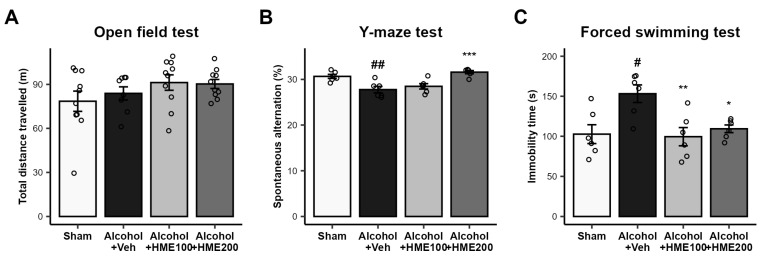
The effect of HME on the locomotor activity of alcohol-ingested mice during behavioral testing. (**A**) The effect of HME on the locomotor activity of the mice was determined by the open field test (OFT). (**B**) The effect of HME on spontaneous alteration in alcohol-ingested mice in the Y-maze test. (**C**) The effect of HME on depression-like behaviors as examined by immobility in the forced swimming test. The values are represented as mean ± SEM. n = 6–11/group. ^#^ *p* < 0.05, ^##^ *p* < 0.01, compared with sham group; * *p* < 0.05, ** *p* < 0.01, *** *p* < 0.001, compared with Alcohol + Veh group. Sham, sham control; Alcohol + Veh, EtOH administration and vehicle treatment; Alcohol + HME100, EtOH administration and HME 100 mg/kg treatment; Alcohol + HME200, EtOH administration and HME 200 mg/kg treatment. EtOH, ethanol; HME, *Heracleum moellendorfii Hance* extract; Veh, vehicle.

The OFT was performed to determine whether the baseline motility of the experimental animals was normal. There was no significant difference in the motility of the mice among the four groups (sham, alcohol + vehicle, alcohol + HME100, and alcohol + HME200). This means that alcohol treatment for eight weeks did not affect the motor performance of the mice, and the substances did not affect the behavioral test results (Figure 2A).

### 2.3. Effect of HME Treatment on Spontaneous Behavioral Changes in Alcohol-Ingested Mice in the Y-Maze Test

The Y-maze test is a behavioral experiment used to assess learning and spatial working memory in mice. The Alcohol + Veh group showed decreased spontaneous alternation compared to the sham group. However, the Alcohol + HME200 group showed increased spontaneous alternation, and the result was almost similar to that of the sham group. In the Alcohol + HME100 group, the behavior was not much different from that of the alcohol-ingested control group and the Alcohol + Veh group (Figure 2B).

### 2.4. Effect of HME Treatment on Depression in Alcohol-Ingested Mice in the Forced Swimming Test

As an affective test for alcohol-induced depression, the FST was conducted to investigate the depression status in the experimental animals. The Alcohol + Veh group showed significantly longer immobility time, indicating increased depression-like behaviors. HME treatment at both doses reversed the immobility time in alcohol-treated mice. Active motility was observed in the Alcohol + HME100 and Alcohol + HME200 groups, similar to that in the sham group (Figure 2C).

### 2.5. Effect of HME Treatment on BDNF Expression in the Hippocampus of Alcohol-Ingested Mice

**Figure 3:**
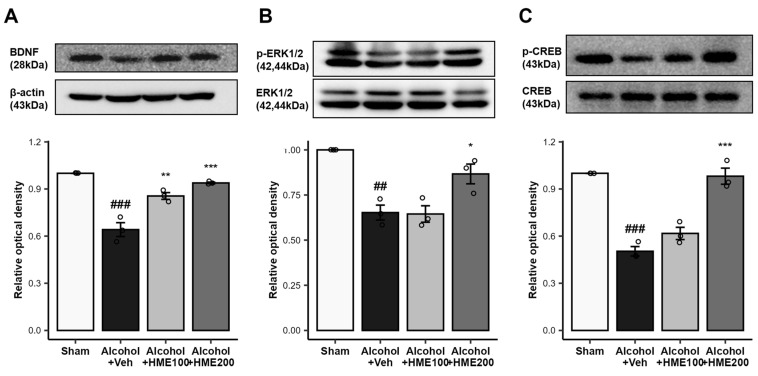
The effect of HME treatment on synaptic plasticity-related protein expression in the hippocampus of alcohol-ingested mice. (**A**) The effect of HME treatment on BDNF expression in the hippocampus. HME treatment upregulated the expression of BDNF in the hippocampus of alcohol-ingested mice. Representative immunoblot for BDNF and quantified results normalized to β–actin expression in hippocampus extracts. (**B**) The effect of HME treatment on p-ERK1/2 expression in the hippocampus of alcohol-ingested mice. HME treatment increased the phosphorylation of ERK1/2 in the hippocampus of alcohol-ingested mice. The representative immunoblot for ERK1/2 and quantified results normalized to β–actin expression in hippocampus extracts. (**C**) The effect of HME on p-CREB expression in the hippocampus of alcohol-ingested mice. HME treatment increased the phosphorylation of CREB in the hippocampus of alcohol-ingested mice. The representative immunoblot for CREB and quantified results normalized to β–actin expression in hippocampus extracts. The values are the mean ± SEM. n = 3/group. ^##^ *p* < 0.01, ^###^ *p* < 0.001, compared with sham group; * *p* < 0.05, ** *p* < 0.01 compared with Alcohol + Veh group. *** *p* < 0.001 compared with Alcohol + Veh group. Sham, sham control; Alcohol + Veh, EtOH administration and vehicle treatment; Alcohol + HME100, EtOH administration and HME 100 mg/kg treatment; Alcohol + HME200, EtOH administration and HME 200 mg/kg treatment; BDNF, brain-derived neurotrophic factor; CREB, cAMP response element binding protein; ERK1/2, extracellular signal regulated kinase; EtOH, ethanol; HME, *Heracleum moellendorfii Hance* extract; Veh, vehicle.

BDNF is a neurotrophic factor that is associated with synaptic plasticity and regulates memory [7]. After eight weeks of alcohol intake, BDNF levels were significantly decreased compared to the sham (*p* < 0.001). Treatment with 100 mg/kg and 200 mg/kg HME upregulated the expression of BDNF compared to the Alcohol + Veh group (*p* < 0.01 and *p* < 0.001, respectively; Figure 3A).

### 2.6. The Effect of HME Treatment on the Phosphorylation of ERK1/2 in the Hippocampus of Alcohol-Ingested Mice

ERK1/2 is known to be involved in memory processes and social and emotional behaviors in adulthood, and ERK1/2 directs the migration of neurogenesis and the eventual fate of neural progenitors during embryonic development [24]. Here, prolonged alcohol exposure reduced the phosphorylation of ERK1/2, which was significantly lower than that in the sham group (*p* < 0.01). HME 200 mg/kg treatment activated the phosphorylation of ERK1/2, whereas HME 100 mg/kg treatment did not (Alcohol + HME100, *p* > 0.05; Alcohol + HME200, *p* < 0.05; Figure 3B). 

### 2.7. The Effect of HME Treatment on the Phosphorylation of CREB in the Hippocampus of Alcohol-Ingested Mice

The phosphorylation of CREB, which plays a critical role in memory formation, serves as a molecular marker of memory processing in the hippocampus responsible for spatial learning [25]. The level of phosphorylation of CREB was significantly lower in the Alcohol + Veh group than in the sham group (*p* < 0.001). HME 200 mg/kg treatment increased the level of phosphorylation of CREB in the hippocampus of alcohol-treated mice compared to the Alcohol + Veh group (*p* < 0.001). HME 100 mg/kg treatment did not change the phosphorylation of CREB in alcohol-treated mice (*p* > 0.05; Figure 3C).

### 2.8. The Effect of HME Treatment on Lipid Accumulation in the Livers of Alcohol-Ingested Mice

**Figure 4:**
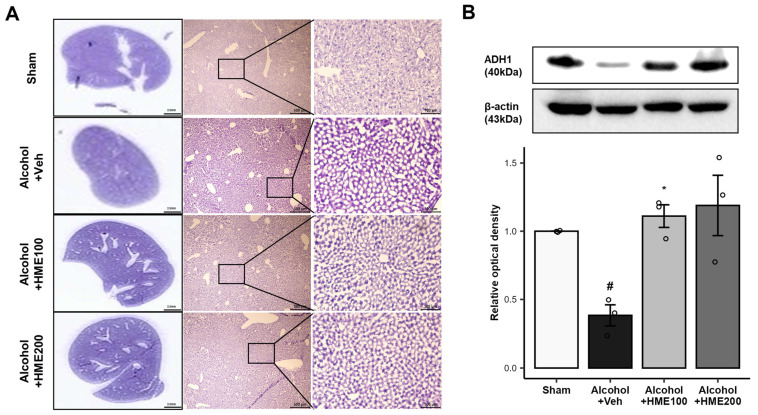
The effect of HME on alcohol-induced damage in the livers of alcohol-ingested mice. (**A**) The effect of HME on alcohol-induced injury in liver tissue. HME treatment reduced lipid accumulation in the livers of the alcohol-ingested mice. The representative morphological images of the livers with H&E staining are as shown. (**B**) The effect of HME on ADH1 expression in the liver. HME treatment increased ADH1 in the livers of the alcohol-ingested mice. The representative immunoblot for ADH1 and quantified results normalized to β-actin expression in hippocampus extracts. The values are the mean ± SEM. n = 3/group. # *p* < 0.05, compared with sham group; * *p* < 0.05 compared with Alcohol + Veh group. Sham, sham control; Alcohol + Veh, EtOH administration and vehicle treatment; Alcohol + HME100, EtOH administration and HME 100 mg/kg treatment; Alcohol + HME200, EtOH administration and HME 200 mg/kg treatment; ADH1, alcohol dehydrogenase 1; EtOH, ethanol; HME, Heracleum moellendorfii Hance extract; Veh, vehicle.

Fatty livers were identified by staining liver tissue to determine the degree of inflammation and hepatocyte damage caused by alcohol-induced toxicity. The histologic morphology of the liver tissues is shown in Figure 4A. Prolonged exposure to alcohol results in lipid vacuolation. HME dose-dependently reduced lipid vacuolation induced by long-term alcohol exposure (Figure 4A).

### 2.9. Effect of HME Treatment on ADH1 Expression in the Liver of Alcohol-Ingested Mice

ADH1 levels were measured in each group to assess the detoxification ability of alcohol-induced toxicity [10]. Alcohol-induced toxicity inhibited the expression of ADH1 in the liver tissue (*p* < 0.05). Conversely, the HME treatment groups, the Alcohol + HME100 group and the Alcohol + HME200 group, showed an increased expression of ADH1, implying that HME treatment restored lipid metabolism (Alcohol + HME100, *p* < 0.05; Alcohol + HME200, *p* < 0.05; Figure 4B).

## 3. Discussion

The purpose of this study was to investigate the beneficial effects of HME on alcohol-induced changes in cognition, mood, and alcohol metabolism. Behavioral tests were used to assess memory and depression. BDNF and the phosphorylation of ERK1/2 and CREB in the hippocampus, lipid vacuolation, and ADH1 in the liver were experimentally assessed in mice treated with 20% EtOH.

The experimental design involved administering 10% ethanol for the first 3 weeks, followed by 20% ethanol for the subsequent 2 weeks and maintaining 20% ethanol for the final 3 weeks. This approach gradually increased and sustained high ethanol intake, similar to methodologies in previous chronic alcohol consumption models. Most research designs for studying chronic alcohol effects on cognitive decline use non-voluntary models [26]. Our approach adapts the chronic ethanol feeding plus multiple binge models, such as the chronic NIAAA model [27,28]. Chronic ethanol feeding models typically last 4–6 weeks, sometimes extending to 12 weeks [27]. Models assessing cognitive function usually increase alcohol concentration gradually over 3–4 weeks [29,30]. This gradual increase mitigates initial aversion and avoids sudden adverse effects, ensuring stable intake for consistent cognitive and physiological outcomes.

The efficacy of the HME on memory deterioration, depression, and alcohol toxicity in mice induced by alcohol for 7 weeks was not sufficient at low doses but effective at high doses. Treatment with HME 100 mg/kg or 200 mg/kg did not affect the mean body weight or mean liver weight. There were no significant differences in locomotor activity between the four groups according to the OFT. The Y-maze and FST were performed to assess cognition and mood changes. In the spontaneous alternation in Y-maze test to evaluate spatial short-term memory evaluation in mice, memory improvement was observed in mice treated with HME at 200 mg/kg but not at 100 mg/kg. In the FST to evaluate depression, both 100 mg/kg and 200 mg/kg HME showed significant differences to the Alcohol + Veh group, which were almost similar to the sham group. This finding suggests that HME may reduce alcohol-induced depression. In the Alcohol + Veh group, there was less mobility, which is indicative of depressive symptoms. Patients with alcohol-induced memory impairment are particularly susceptible to depression because depressive symptoms and cognitive impairment are closely related to damage to neuroplasticity, as evidenced by reductions in BDNF, ERK1/2, and CREB [31]. Considering the epidemiology of ARD as secondary dementia, they are more likely to have a history of alcohol abuse [32]. Patients with ARD are susceptible to depression in their past history, and the repetitive consumption of alcohol increases the risk of developing depression. Controlling depression is another important requirement for the development of novel interventions for ARD [33]. The two most significant risk factors for early-onset dementia are alcohol consumption and depression, which are also modifiable risk factors for late-onset dementia [34,35]. Thus, HME may be a potential adjuvant treatment for depression, which often accompanies ARD.

Consistent with the results of the behavioral studies, Western blotting showed that HME strongly supports the prevention and treatment effects of HME against alcohol-induced cognitive decline by activating the protein expression of BDNF and phosphorylation of ERK1/2 and CREB in hippocampal tissues, as shown in the HME 200 mg/kg group. The enhanced expression of BDNF, p-ERK1/2 and p-CREB in the hippocampus of mice suffering from alcohol-induced cognitive decline and depression demonstrated the efficacy and potential of high-dose HME treatment as a preventive and therapeutic medicine for ARD.

ARD and alcohol-induced cognitive impairment are associated with structural and functional brain damage, particularly in the domains of visuospatial function, memory, and executive tasks [14]. BDNF has been implicated in the identification of neurocognitive function in individuals with alcohol dependence [36]. A recent meta-analysis revealed that alcohol consumption was associated with a significant decrease in BDNF blood levels and alcohol withdrawal showed a non-significant trend for increasing BDNF levels, with significant increases observed with longer withdrawal periods [37]. Ethanol exposure resulted in a downregulated ERK signaling pathway in the prefrontal cortex and the hippocampus with decreased mRNA levels of the neurogenic factors such as *Mki67*, *Sox-2*, *Dcx, Ncam* and *Calb1* [6,38,39]. Several studies revealed that hippocampal BDNF-CREB signaling pathway was responsible for reversing the memory deficit caused by alcohol exposure [5,40]. The BDNF-ERK-CREB pathway is important for memory formation and synaptic plasticity [41]. Its disruption causes a cognitive decline in Alzheimer’s disease, and studies have suggested that it may be a promising target for therapy [41]. Given the effective increase in the expression of BDNF, ERK1/2 and CREB in the treatment group in the alcoholic cognitive impairment mouse model, HME is expected to improve cognitive function and depression through neuronal recovery and regeneration.

Chronic alcohol intake induces liver injury and neuroinflammation in the hippocampus and prefrontal cortex, which impairs memory and cognition in correlation with inflammatory cytokines such as tumor necrosis factor-α (TNF-α), interleukin-6 (IL-6), monocyte chemoattractant protein-1 (MCP1), and interleukin-1β (IL-1β), suggesting a liver–brain axis contributing to cognitive decline [5,28]. Alcohol-induced lipid accumulation in the liver results from the chronic or binge consumption of alcohol [42].

Mice treated with HME showed the least amount of lipid accumulation and also had a beneficial effect on ethanol metabolism. The alcohol + HME200 group almost reversed the liver tissue profiles of the sham group. Considering that the main cause of fatty liver is alcohol consumption and there was no confounding factor such as other drug treatment or a hyperlipidemia-induced diet, the main cause of fatty liver in this experiment is attributed to drinking [43]. Even though the Alcohol + Veh group weighed the least, their liver weight was the highest, and the proportion of lipids in the liver tissue was the highest among the four groups. These changes indicate that alcohol ingestion is the main cause of liver damage. As confirmed by H&E staining of the liver tissues, less lipid accumulation was observed in the HME-treated groups than in the alcohol-treated group. This suggests that HME may prevent and treat alcohol-induced fatty liver. Furthermore, ADH1, an alcoholic dehydrogenase, was the highest in the Alcohol + HME200 group. The activity of ADH1 in the liver is considered the primary factor associated with systemic alcohol metabolism and it contributes to approximately 70% of alcohol metabolism [44]. Alcohol is converted to acetaldehyde by ADH1 in the liver, and then and is transported to each organ including the brain [45,46]. When ADH-deficient mice undergo chronic alcohol feeding, they exhibit a 14-fold increase in blood EtOH concentration and a 1.5-fold increase in acetaldehyde concentration compared to ADH-normal mice [47]. The findings suggest that the presence of ADH reduces the burden of alcohol on other organs in the body. Blood EtOH and acetate, the consequent metabolites of acetaldehyde processed by aldehyde dehydrogenase (ALDH), enter astrocytes and neurons and undergo cellular ethanol metabolism in the brain [48]. This indicates that a high dose of HME may protect against liver damage by enhancing alcohol metabolism in the liver and improve alcohol-induced cognitive impairment in the brain. This ingredient is beneficial for patients with ARD who are prone to liver dysfunction because it protects both organs.

These findings may be related to previous studies on identified bioactive compounds and their biological effects. Skimmin exhibited an inhibitory effect on human MAO-A and MAO-B enzymes [49]. It also has antioxidant and anti-inflammatory activities, inhibiting inflammatory factors like TNF-α, IL-1β, and IL-6, and inactivating proinflammatory signaling pathways such as STAT3, STAT1, and ERK1/2. Additionally, skimmin decreased the levels of malondialdehyde (MDA) and increased the levels of glutathione (GSH) and superoxide dismutase (SOD) [50]. Isopimpinellin inhibited hippocampal neuron apoptosis, increased GABA levels, and upregulated genes related to GABA and 5-HT receptors [51]. Additionally, it has been demonstrated to have protective effects against epilepsy [52,53]. Further studies are needed to elucidate the detailed mechanisms underlying its protective effects on neurons and to gather more information about the other ingredients of HME.

This study has several limitations. First, when establishing an experimental animal model of alcohol-induced cognitive impairment, alcohol is usually not forcibly injected into mice. In this study, alcohol was forcibly injected immediately before the behavioral experiment to induce binge drinking. Although we attempted to create a model that most closely resembles human drinking in everyday life, the environment in which only alcohol is provided instead of water may cause the experimental animals to voluntarily refuse to consume fluids. Despite the monitoring of fluid consumption during the study, there is a possibility of inconsistent alcohol consumption among the experimental animals. The results of this study must be interpreted with caution and the change in methodology for establishing an animal experimental model of cognitive impairment must be considered [26]. Since the experiment relied to some extent on voluntary alcohol consumption by the mice, it could not serve as a model for severe alcoholic dementia. Second, it is acknowledged that the OFT was not conducted prior to grouping, which would have ensured the fairness and comparability of the groups. Third, assessing cognition and depressive behavior through a single test provides limited evidence. A more comprehensive assessment with multiple tests would enhance credibility. Furthermore, binge drinking before the behavioral tests might have made it difficult to distinguish between alcohol-induced cognitive impairment and the direct effects of alcohol. Fourth, for the further validation and analysis of alcohol-induced hepatotoxicity, not only ADH1 but also aldehyde dehydrogenase, which decomposes acetaldehyde, a substance that causes toxicity after alcohol decomposition, should be included in the evaluation [54]. In addition, the degree of lipid accumulation in the liver could be more accurately measured by other methodologies, such as gas chromatography and low-field nuclear magnetic resonance. Fifth, the detailed mechanisms and pharmacokinetics of the major compounds in HME warrant further investigation. It is necessary to explore other potential mechanisms of action, such as the effects of HME on oxidative stress and inflammatory pathways in both the liver and brain. These aspects are crucial for a more comprehensive understanding of HME’s therapeutic potential and its role in mitigating alcohol-induced damage.

## 4. Materials and Methods

### 4.1. Preparation of Heracleum moellendorffii Hance Root Extract (HME)

HME was obtained from Yeongwol, Korea. A dried voucher specimen is deposited in the herbarium of the Department of Korean Neuropsychiatry at Kyung Hee Medical Center (register number KH-019). Dried roots of HME (600 g) were boiled twice for two hours with 30% ethanol, and the extract was filtered using filter paper. After filtration, the extract was concentrated using a rotary evaporator. The decoction was then lyophilized to obtain a brown powder. The total yield of dried extract from the original natural product was approximately 8% (*w*/*w*). The powder was stored at 4 °C until the start of the experiment.

**Figure A1:**
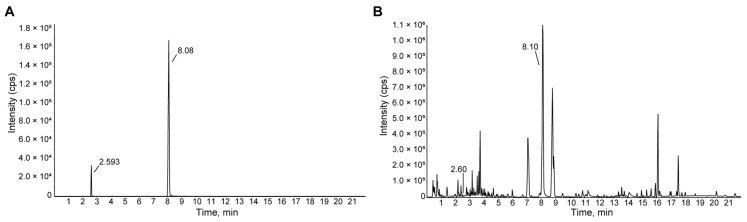
Results of the base peak chromatogram (BPC) of skimmin and isopimpinellin from (**A**) reference standards and (**B**) the 30% ethanol extract of HME. A peak at 2.60 corresponds to skimming and a peak of 8.10 corresponds to isopimpinellin.

The marker compounds, skimmin and isopimpinellin, were detected in the HME to ensure its quality. The identification of skimmin and isopimpinellin found in HME was performed using ultra-performance liquid chromatography triple time-of-flight mass spectrometry/mass spectrometry (UPLC-Triple TOF-MS/MS). The analysis was performed using the Thermo Scientific Vanquish UHPLC system (Thermo Fisher Scientific, Sunnyvale, CA, USA) and the Triple TOF 5600+ mass spectrometer system (Triple TOF MS; QTOF, Sciex, Foster City, CA, USA). The analytical level used for the qualitative analysis was 50 mg/mL. The reference standards, skimmin (CAS 93-39-0, RS877545) and isopimpinellin (CAS 482-27-9, RS888768), were purchased from Interpharm Inc. (Goyang, Republic of Korea). The results of the compounds found in the 30% ethanol extract of HME by UHPLC are shown in Figure A1. The chromatographic analysis, conducted using the UHPLC system, revealed peaks for skimmin at 2.60 min and isopimpinellin at 8.10 min, thereby verifying the presence of these marker compounds in HME.

### 4.2. Animals

C57BL/6 mice (7-week-old males, 20–22 g) purchased from Orient Bio Inc. (Seongnam, Republic of Korea) were acclimated for 1 week at the animal center of Kyunghee University Hospital. Mice were maintained at a constant temperature (22 ± 2 °C) and relative humidity (60 ± 10%). Mice were housed in acrylic cages (20 × 27 × 12 cm), with free access to food, under an artificial 12 h light/dark cycle (lights off at 18:00). All behavioral tests were performed between 10:00 and 17:00. The experiment was conducted according to the regulations of the Kyung Hee University Medical Center Institutional Animal Care and Use Committee (Approval No. KHMC-IACUC 16-033).

Forty mice were randomly divided into four groups of 10 mice each. The four groups were as follows:

(1) Sham: unlimited access to food and water without alcohol;
(2) Alcohol + Veh: unlimited access to food and EtOH without water + 0.2 mL of distilled water as the vehicle treatment;
(3) Alcohol + HME100: unlimited access to food and EtOH without water + HME 100 mg/kg treatment (low dose);
(4) Alcohol + HME200: unlimited access to food and EtOH without water + HME 200 mg/kg treatment (high dose).

The sham group of mice was treated with alcohol-free water and reared on ad libitum feeding. The alcohol +Veh group and the two HME-treated groups were voluntarily given 10% ethanol instead of water for the first 3 weeks, and 20% EtOH instead of water for the subsequent 2 weeks, to increase their alcohol intake. For the next 3 weeks, 20% EtOH was used instead of water for maintenance. At the same time, the alcohol + HME100 group received 100 mg/kg of HME daily via gavage, and the alcohol + HME200 group received 200 mg/kg of HME daily via gavage. Immediately before each behavioral test, binge drinking was induced by an oral injection of 1.5 g/kg of 20% EtOH.

**Figure 5:**
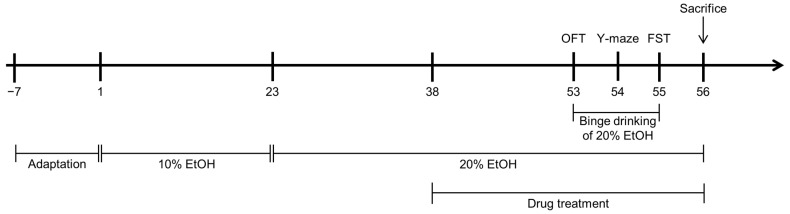
Experiment schedule.

The experimental schedule is illustrated in Figure 5. The body weights of the mice in the four groups were measured three times a week for 7 weeks, and the mean weight was calculated. The weights were also normalized to their baseline weights at the start of the experiment to account for individual differences. The mean weekly alcohol consumption was also measured.

### 4.3. Open Field Test (OFT)

To evaluate the locomotor activity in mice, we performed an open field test (OFT), based on previous studies [55,56]. Each mouse was individually placed on a white square plexiglass apparatus (50 × 50 × 50 cm) in a dimly lit room, followed by video tracking for 10 min. Locomotor activity was analyzed based on the movement distance in the total grids using SMART (PanLab Co. Barcelona, Spain), a computerized image tracking analysis program.

### 4.4. Y-Maze Test

The Y-maze test was performed to assess short-term working memory (Y-arm length, 40 cm; bottom arm width, 4 cm; wall height, 12 cm) [57]. Mice were placed in one arm and allowed to move freely for 8 min. The number of entries into each arm was then counted. Alternation refers to a series of entries into three arms. Spontaneous alternation behavior (%) was calculated as the ratio of the number of actual alternations to the total number of possible alternations as follows: the total number of possible alternations was equal to the number of arm entries minus two.

$$Spontaneous\;alternation\;behavior\;\left(\%\right)=\frac{Number\;of\;alternations}{\left(Total\;number\;of\;arm\;entries-2\right)}\times 100$$

### 4.5. Forced Swimming Test (FST)

To evaluate depressive-like behaviors, a forced swimming test was performed [58]. Each mouse was individually placed in a transparent cylindrical glass cylinder (height: 46 cm, diameter: 20 cm) with a water depth of 15 cm and temperature of 23–25 °C. Opaque baffles were placed between the cylinders to prevent the mice from seeing each other. The activity of the mice was monitored for 6 min. Immobility was recorded only during the last 4 min of the total 6 min, and the mice were acclimatized to swim during the first 2 min out of the 6 min. Mice were considered immobile when they stopped struggling and made only small movements necessary to float or keep their head above the water.

### 4.6. Tissue Preparation

After the behavioral tests were completed, the animals were sacrificed. For Western blotting, the hippocampus was dissected. For hematoxylin and eosin (H&E) staining, mice were perfused with phosphate-buffered saline (PBS) and 4% paraformaldehyde solution. Whole brains and livers were isolated and postfixed in 4% paraformaldehyde for 24 h, at 4 °C, and then transferred to 20% sucrose solution. Each specimen was embedded in an optimal cutting temperature (OCT) compound. Tissues were stored at −70 °C until analysis.

### 4.7. Western Blotting

Western blotting was performed on the isolated mouse brain tissue. Hippocampal tissue was homogenized in 1× RIPA buffer (Pierce Biotechnology, Rockford, IL, USA). Lysates containing protease inhibitors and phosphatase inhibitors were centrifuged at 12,000× *g* for 15 min at 4 °C, and the supernatant fluid was collected. Proteins were quantified using a BCA protein assay kit (Pierce Biotechnology, Rockford, IL, USA). The extracted proteins were electrophoresed on a 12% SDS-PAGE gel and transferred to a polyvinylidene fluoride (PVDF) transfer membrane (Millipore, Billerica, MA, USA) for 1 h. The membrane was blocked with 5% nonfat skim milk in TBST for 1 h at room temperature. BSA (5%) in TBST was used as the phospho-form antibody. The membrane was incubated with primary antibodies in a blocking buffer overnight at 4 °C. Primary antibodies used were as follows: anti-ERK1/2 rabbit antibody, anti-p-ERK1/2 rabbit antibody (1:2000, Cell Signaling Technology, Beverly, MA, USA), anti-BDNF rabbit antibody (1:200, Alomono Labs, Jerusalem, Israel), anti-CREB rabbit antibody, anti-p-CREB rabbit antibody (1:1000, Cell Signaling Technology, Beverly, MA, USA), anti-alcohol dehydrogenase1 (ADH1) rabbit antibody (1:1000, Cell Signaling Technology, Beverly, MA, USA), and anti-β-actin mouse antibody (1:10,000, Santa Cruz Biotechnology, Santa Cruz, CA, USA). After washing three times with TBST for 10 min each, the membrane was incubated with HRP-conjugated secondary antibodies in a blocking buffer for 1 h at room temperature. The secondary antibodies used were goat anti-Mouse IgG (H + L) secondary antibody (1:10,000, Invitrogen, Carlsbad, CA, USA) and goat anti-Rabbit IgG (H + L) secondary antibody (1:10,000, Invitrogen, Carlsbad, CA, USA). After washing three times with TBST for 10 min, the membranes were developed using Davinch-Chemi^TM^ (Celltagen, Seoul, Republic of Korea). Immunoreactive bands were detected using an enhanced chemiluminescence kit (Pierce Biotechnology, Rockford, IL, USA). Quantification was performed using the ImageJ v.1.44 software (NIH, Bethesda, MD, USA).

### 4.8. Histological Examination of the Liver Tissues

Liver tissues were sectioned at 10 μm sections using a cryostat microtome (Leica CM1850; Leica Microsystems, Wetzlar, Germany) and the slices were placed on slides. After washing with PBS, the sections were subjected to hematoxylin & eosin (H&E) staining. Microscopic analysis was performed with a BX51 microscope (Olympus, Tokyo, Japan).

### 4.9. Statistical Analysis

The results of this study are presented as the mean ± standard error of the mean (SEM). One-way analysis of variance (ANOVA) followed by Bonferroni’s post hoc test was used for the analysis. Data were analyzed using the R ver 4.1.2 software [59]. *p* < 0.05 was considered statistically significant.

## 5. Conclusions

In conclusion, the alcohol + HME200 group was treated with a high dose of HME, which showed beneficial effects on alcohol-induced memory loss and cognitive decline related to chronic alcohol consumption for eight weeks, as assessed by the Y-maze test and FST. HME treatment improved BDNF, p-ERK1/2, and p-CREB. In addition, HME decreased lipid accumulation in the liver and increased the level of ADH1. Our findings confirmed that HME may be useful as a potential preventive and therapeutic drug for the symptoms of alcohol-induced dementia by enhancing the effect of neural plasticity and neutralizing the effects of alcohol-induced toxicity. The findings of this research indicate that natural compounds may be employed as alternative therapies for the treatment of alcohol-induced cognitive impairments, which are increasing in prevalence but currently lack standard treatments.

## Data Availability

Data used to support the findings of this study are available from the corresponding author upon reasonable request.
